# Botulinum toxin-A: evaluation of the influence on the aspect of trunk scars^☆^^☆☆^

**DOI:** 10.1016/j.abd.2020.06.024

**Published:** 2021-03-24

**Authors:** Renato Luiz Baldissera, Rafaela Ludvig Lehmkuhl, Juliano Vilaverde Schmitt, Deborah Skusa de Torre

**Affiliations:** a Department of Dermatology, Hospital Universitário Evangélico de Curitiba, Curitiba, PR, Brazil; b Department of Dermatology, Universidade Estadual Paulista, Botucatu, SP, Brazil

Dear Editor,

During tissue healing, fibroblasts differentiate into myofibroblasts, which play a crucial role in wound contraction. After the wound closes, myofibroblasts undergo apoptosis. Mediated by several factors, such as mechanical stress and TGF-B1 expression, myofibroblast survival can be stimulated with the development of hypertrophic scars. It is believed that botulinum toxin A (BTA) directly inhibits fibroblast-myofibroblast differentiation, suppresses TGF-B1 expression, and inhibits the release of inflammatory mediators that could prevent scar hypertrophy, indicating its potential preventive use in scars located in areas subject to tension.^1^, ^2^, ^3^, ^4^, ^5^ The aim of the study was to evaluate the influence of BTA use in the immediate postoperative period after the excision of melanocytic nevi on the trunk, aiming to prevent unaesthetic scars.

This was a prospective study of therapeutic intervention, and the project was approved by the institutional Research Ethics Committee of the Evangelical College of Paraná (n. 1,162,242). The participants signed the Free and Informed Consent form. The inclusion criteria consisted of age over 12 years and diagnosis of melanocytic nevus on the trunk with a diameter ranging between 0.7 and 1.4 cm. Exclusion criteria consisted of healing defects, coagulopathies, pregnancy/breastfeeding, autoimmune disease, and smoking. A total of 23 lesions (n  = 23) were selected from 16 participants, 9 females (n = 15) and 7 males (n = 8), aged 16 to 80 years, skin phototypes II to IV, subdivided into five regions: anterior thorax (n = 2), posterior cervical (n = 2), upper back (n = 11), lower back (n = 6) and deltoid (n = 2) areas (Table 1).

**Table 1:** Table with the main data of the participants and the results obtained.

Location	Patient	Sex	Age	Largest diameter (cm)	Enlarged scar	Difference in width >1 mm
Thorax	A	Male	17	1.1	Yes	No
	B	Female	35	0.7	Abandoned the protocol	
Posterior cervical region	C	Female	33	0.8	Yes	Yes
	D	Female	50	1	No	Yes
Upper back	B	Female	35	0.7	Abandoned the protocol	
	E	Female	34	0.7	Yes	No
	F	Female	77	0.7	No	No
	B	Female	35	0.8	No	No
	G	Male	66	0.8	No	No
	H	Male	65	0.8	No	No
	I	Female	17	0.8	Yes	No
	J	Male	48	0.9	No	No
	K	Male	57	1	No	Yes
	L	Male	44	1	Yes	No
	M	Female	24	1.4	Yes	No
Lower back	B	Female	35	0.7	Abandoned the protocol	
	N	Female	35	0.7	Postoperative infection	
	I	Female	17	0.8	Yes	No
	C	Female	33	0.9	Yes	No
	O	Male	18	0.9	Yes	No
	P	Female	39	1.1	Yes	No
Deltoid	P	Female	59	1.1	Yes	No
	O	Male	18	0.9	Yes	No

The lesions were marked as a 2-cm ellipse, following Langer’s lines, encompassing the nevus with a 2-mm lateral margin, resulting in spindles of the same length and varying width, according to the nevus diameter. Preoperative asepsis was performed with iodine-povidone, perilesional application of lidocaine anesthetic with vasoconstrictor in equal volume for all nevi, followed by incision, excision, detachment of the borders, internal suture with polyglactin (Vicryl 4.0 Ethicon®) at 3 fixed equidistant stitches and external suture with 4.0 mononylon (Ethicon®) at 4 stitches 2-mm equidistant from the borders. Immediately after the suture, 4 stitches were marked for intradermal application of 1 Unit (U) per stitch of BTA (total of 4U) on one side of the incision line. Incobotulinum toxin A/ Xeomin® 100U (Merz Farmacêutica), diluted in 1 mL of 0.9% saline solution was used. When the incision direction was longitudinal, BTA was applied to the half at the surgeon’s right and when vertical, to the upper half (Fig. 1). The participant was not informed about which half received the BTA. The excised nevi were sent for histopathological analysis. The stitches were removed after 14 days. The participants were followed for six months, and clinical reevaluation and photographic records were performed after two and six months.

**Figure 1:**
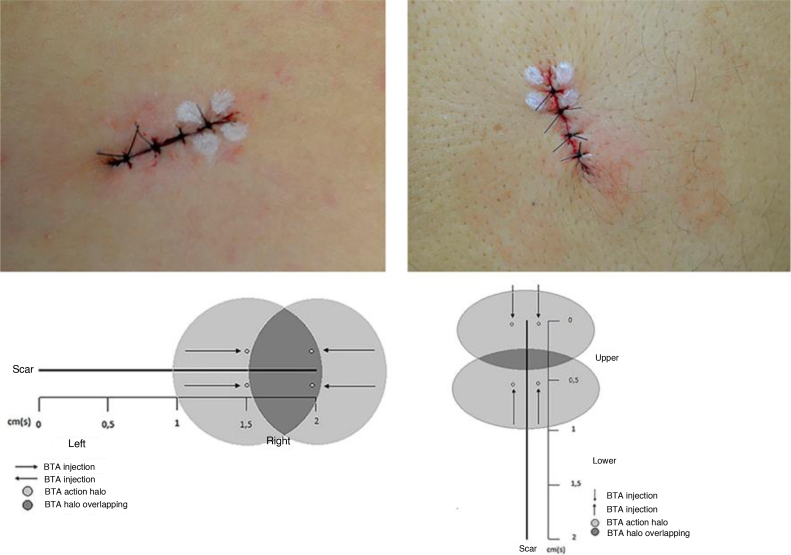
Marking of BTA stitches with a schematic representation of the proposed methodology.

The evaluation parameters of the results were inspection of the scars, comparison between the two halves, measurements, and the participants’ opinion. An enlarged scar was considered when the final width was greater than 50% of the initial measurement, that is, of the spindle before suturing, corresponding to the nevus diameter plus 2 mm of margin. Scars with a width lower than this parameter or with complete coaptation of the borders were considered not enlarged. For the comparison between the halves, the result was considered when the difference was greater than 1 mm, as long as the half treated with BTA had a smaller width. The participants’ opinions were evaluated regarding the width, thickness, and texture between the scar halves.

Continuous variables were submitted to bivariate analysis using the parametric Student’s *t* test and the non-parametric Mann-Whitney test, depending on the normality of the distributions, assessed by the Shapiro-Wilk test. Categorical variables were compared by Fisher’s exact test. The 95% Confidence Interval of the proportions was calculated using the bootstrap method. Values of p <0.05 were considered significant.

Fourteen of 16 patients completed the study, 7 women (n = 10) and 7 men (n = 8), with a mean age of 43.5 years (17–77 years, SD = 19.29), totaling 18 surgical scars (n = 18). The mean diameter of the nevi was 0.92 cm. Enlargement was observed in 12/18 (66.67%) scars (95% CI: 44.5% –83.3%). One 18-year-old participant had a keloid scar in the deltoid region. A smaller width was measured in the BTA-treated half (Fig. 2) in 3/18 (16.7% [95% CI: 0% –33%]) scars, two located on the posterior cervical and one on the upper back regions. None of the scars showed a smaller width in the control half than the BTA-treated half. The risk of scar enlargement was not correlated with the nevus diameter (p = 0.307), but it was inversely associated with age, being five times lower for participants aged 50 years or older (RR = 0.2 [0, 05–0.74]; p = 0.013). In the participants’ opinion, only 4/14 (22.2% [95% CI: 5.6% –33.3%]) noticed differences between the halves, considering an improvement in the treated half.

**Figure 2:**
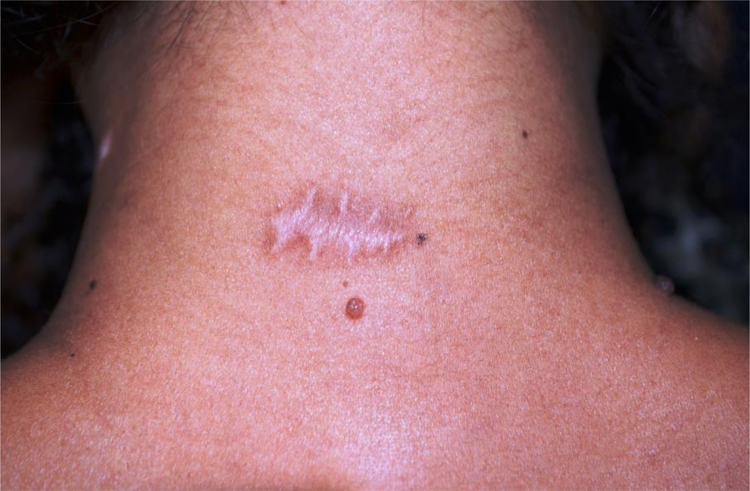
Final aspect with smaller width and less raised scar tissue on the side treated with BTA at the end of the follow-up.

In this study, the intradermal injection of the Incobotulinum toxin A in the immediate postoperative period and at the dose used was not able to promote significant changes in the quality of the scars.

## Financial support

A bottle of Incobotulinum toxin A (INCO)/Xeomin® 100U (Merz, Brazil) was donated, without any conflicts of interest, that is, the company did not interfere in any stage of the study and the writing of this manuscript.

## Authors’ contributions

Renato Luiz Baldissera: Conception and planning of the study; preparation and writing of the manuscript; data collection, analysis, and interpretation; intellectual participation in the propaedeutic and /or therapeutic conduct of the studied cases; critical review of the literature.

Rafaela Ludvig Lehmkuhl: Conception and planning of the study; intellectual participation in propaedeutic and/or therapeutic conduct of the studied cases.

Juliano Vilaverde Schmitt: Statistical analysis.

Deborah Skusa de Torre: Conception and planning of the study; effective participation in the research guidance.

## Conflicts of interest

None declared.
